# Extensive exchange of transposable elements in the *Drosophila pseudoobscura* group

**DOI:** 10.1186/s13100-018-0123-6

**Published:** 2018-06-19

**Authors:** Tom Hill, Andrea J. Betancourt

**Affiliations:** 1 The Department of Molecular Biosciences, University of Kansas, 4055 Haworth Hall, 1200 Sunnyside Avenue, Lawrence, KS 66045 USA; 2 Institute of Integrative Biology, University of Liverpool, Liverpool, L69 7ZB UK

## Abstract

**Background:**

As species diverge, so does their transposable element (TE) content. Within a genome, TE families may eventually become dormant due to host-silencing mechanisms, natural selection and the accumulation of inactive copies. The transmission of active copies from a TE families, both vertically and horizontally between species, can allow TEs to escape inactivation if it occurs often enough, as it may allow TEs to temporarily escape silencing in a new host. Thus, the contribution of horizontal exchange to TE persistence has been of increasing interest.

**Results:**

Here, we annotated TEs in five species with sequenced genomes from the *D. pseudoobscura* species group, and curated a set of TE families found in these species. We found that, compared to host genes, many TE families showed lower neutral divergence between species, consistent with recent transmission of TEs between species. Despite these transfers, there are differences in the TE content between species in the group.

**Conclusions:**

The TE content is highly dynamic in the *D. pseudoobscura* species group, frequently transferring between species, keeping TEs active. This result highlights how frequently transposable elements are transmitted between sympatric species and, despite these transfers, how rapidly species TE content can diverge.

**Electronic supplementary material:**

The online version of this article (10.1186/s13100-018-0123-6) contains supplementary material, which is available to authorized users.

## Background

Transposable elements (TE) are parasitic mobile elements that propagate through the genomes of organisms, irrespective of the cost to the host [1–3]. TEs increase their numbers via transposition and the resulting non-Mendelian inheritance, but these factors are countered by suppression of transposition by hosts, by the generation of faulty, inactive copies during transposition, and by purifying selection acting against individual copies of a TE or against a TE family in aggregate [4–7]. Because of these inactivating forces, TEs may go extinct within a lineage, particularly if transposition rates become low due to host suppression [4, 8–10]. TE families can potentially temporarily escape this suppression by invading new hosts, with these horizontal transfers allowing them to persist in spite of extinction within individual lineages [11]. This process is exemplified by the recent horizontal transfer of the *P*-element, newly acquired by *D. melanogaster* sometime in the twentieth century from a Caribbean species *D. willistoni* [12], followed by a further transmission into *D. simulans* [13, 14]. While copies of the *P*-element are typically highly degraded in *D. willistoni*, the element has been recently active in *D. melanogaster* and *D. simulans* [12]. Such horizontal transfer of TEs were once considered rare [12, 15], but have recently been shown to be pervasive, not just in *Drosophila* [5], but in other organisms as well [11, 16–18]. Transfer of TEs are thought to be more common among closely related species, and between those with overlapping geographic ranges [18, 19]. Several transmission events have even been detected between hosts and parasites [19]. Other cases have resulted in changes that reshape the genome or generate phenotypic changes [11, 18], some of which resulted in adaptive changes, or changes involved in domestication [18, 20, 21].

The rates of horizontal transfer has implications for genome evolution. If horizontal transfer is rare, taxonomic groups may diverge in TE content over time, as individual TE families go extinct or are acquired by related species [4, 22–24]. If common, it may maintain active elements through regular exchange of active TEs between species [25]. These factors may go some way toward explaining differences between groups: for example, mammals, have few active TEs with mostly fixed insertions within species [26, 27], while in *Drosophila* TEs are highly active, as inferred from a high proportion of polymorphic insertions [28–32]. The forces of horizontal acquisition and suppression appear to lead to a slow, but detectable rate of turnover in TE content in the *Drosophila* genus: e.g. in the 12-genomes project [33], though all of the sequences species host Long Terminal Repeat (LTR), Long Interspersed Nuclear Elements (LINEs) retroelements and Terminal Inverted Repeat (TIR) DNA transposons [34], the proportion of the genome composed of repeats and the number of families appears to differ between species [33, 34].

Here, we examine the transposable element content in the *D. pseudoobscura* group in the *Sophophora* subgroup of *Drosophila* [33, 35–37]*.* This subgroup consists of four species with largely overlapping ranges, *D. pseudoobscura*, *D. persimilis, D. miranda* and *D. lowei* [37, 38]. *D. pseudoobscura* was initially utilised as a study organism due to patterns of inversion polymorphism and variation in Y chromosome size [39–43]. These species are also able to hybridise to some degree in the lab [44–48], with *D. pseudoobscura* and *persimilis* showing little divergence outside of three fixed inversions between their genomes [45]. Unlike *D. simulans* and *D. melanogaster*, the *D. pseudoobscura* group species are not cosmopolitan [37, 40, 49] and thus may have had less opportunity to encounter new transposable elements due to range expansion or recent ecological changes. We use publicly available genome sequences for the four species, an outgroup species (*D. affinis*), and an improved genome sequence from *D. pseudoobscura* [33, 38, 50]. We use these data to examine changes in TE content among the species, and horizontal transfer of TEs within and from outside this group.

## Results and discussion

### TE annotation of the *D. pseudoobscura* group genomes

**Fig. 1:**
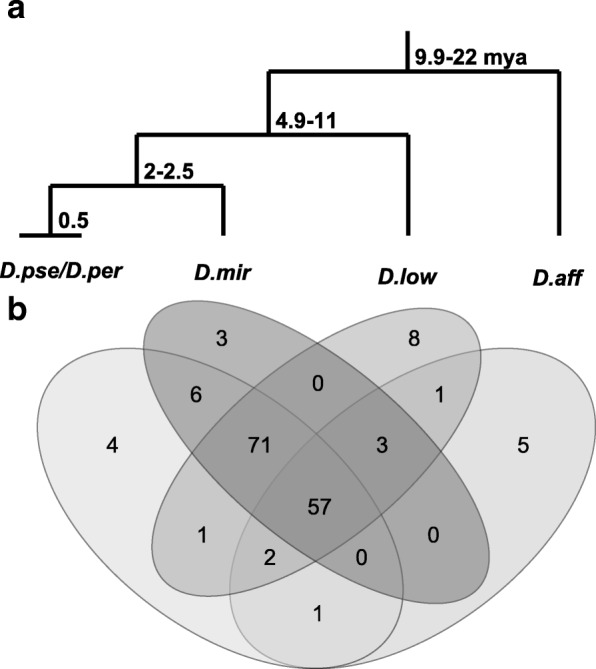
**a** Phylogeny of the *D. pseudoobscura* group species studies here, with labels showing the estimated time of divergence (from [45, 62]). **b** A Venn diagram showing the number of TE families, including putative novel families, shared between the *D. pseudoobscura* group species in **a**

We identified TE families in the genomes of *D. pseudoobscura, persimilis, D. miranda, D. lowei* and *D. affinis*, and manually filtered and curated these sequences to generate 157 well-supported TE families found across the group (Fig. 1, Additional file 1: Figure S1). We also identified 15 sequences that pass all filters, but cannot be assigned to a TE order, these sequences were not included in further analyses (e.g. the 2 unknown sequences in *D. pseudoobscura*, Table 1, Additional file 2: Table S2), though they may represent undescribed TEs. Encouragingly, we found the 116 TE families previously described [51] for *D. pseudoobscura* using our pipeline, showing that our pipeline can independently recover the major families. We also found two TE families known from other *Diptera* species [51], and 28 additional putative TE families belonging to known orders that passed all our filters in these two species.

**Table 1:** TE content of species in the *D. pseudoobscura* group

			Reads			Reference	PPTE2
Species	TE Order	Families	Percent reads	Est. copy number	dnapipeTE (%)	Reference masked (%)	Num. ins
*D. pseudoobscura*	TIR	31	1.745	414	1.65	0.98	292
	LTR	72	8.875	2230	12.67	7.21	1846
	LINE	35	3.633	1121	5.02	2.85	927
	RC	3	1.852	978	2.83	1.21	978
	Polinton	1	0.417	149	0.65	0.081	29
	Unknown	2	0.332	22	0.8	0.017	6
	Total known	142	16.522	4892	22.82	12.33	4072
	Total	144	16.854	4914	23.62	12.5	4078
*D. persimilis*	TIR	31	1.547	413	1.47	1.29	392
	LTR	72	14.273	2260	15.24	12.95	1919
	LINE	35	6.956	1301	6.92	5.76	958
	RC	3	4.43	1781	4.11	3.41	1755
	Polinton	1	0.034	46	0.49	0.18	46
	Unknown	2	0.543	76	0.86	0.025	7
	Total known	142	27.24	5801	28.23	23.59	5070
	Total	144	27.78	5877	29.09	23.62	5077
*D. miranda*	TIR	31	0.892	262	1.85	0.87	258
	LTR	67	7.19	973	10.86	2.21	925
	LINE	36	5.367	1431	9.26	1.25	1059
	RC	5	1.484	1934	2.34	1.16	1934
	Polinton	1	0.054	9	0.03	0.024	9
	Unknown	2	0.337	4	0.27	0.015	4
	Total known	140	14.987	4609	24.34	5.51	4185
	Total	142	15.324	4613	24.61	5.53	4189
*D. lowei*	TIR	31	1.396	495	1.46	0.382	381
	LTR	74	6.883	1366	6.71	1.55	740
	LINE	34	3.839	933	4.03	0.799	449
	RC	5	1.245	813	1.83	0.363	523
	Polinton	1	0.054	7	0.094	0.013	7
	Unknown	9	0.641	265	3.9	0.087	241
	Total known	145	13.417	3614	14.12	3.1	2100
	Total	154	14.058	3879	18.024	3.187	2341
*D. affinis*	TIR	9	0.872	278	3.25	0.177	230
	LTR	47	4.328	630	8.4	1.427	832
	LINE	13	5.223	530	6.4	0.406	339
	RC	4	1.351	369	2.26	0.245	369
	Polinton	1	0.068	35	0.77	0.041	35
	Unknown	10	1.192	206	1.36	0.098	206
	Total known	74	11.842	1842	21.08	2.29	1805
	Total	84	13.034	2048	22.44	2.39	2011

For each species, the table shows the number of TE families annotated for each order, and five metrics of TE content. These are: the proportion of Illumina reads mapping to TE sequences from each order (% reads), the total number of copies from all families of that order, estimated from coverage relative to chromosome 3 (est. copy number), the proportion of the reference genome masked by each order, the proportion of reads (% reads) per TE order as estimated by dnaPipeTE, and the number of insertions found using PopoolationTE2 [90]. As LTR elements often exist not as complete insertions, but as solo-LTRs resulting from illegitimate recombination, coverage for the LTR elements was estimated for both solo LTRs and LTR bodies separately, with the mean taken across the combined sequences. We tested for extrachromosomal circular DNAs such as from Helitrons and Polintons via comparisons between copy numbers and insertion numbers

For *D. pseudoobscura*, we were able to use small RNA and RNAseq data [52, 53] to further support our annotations, particularly for the 28 putatively novel TEs. We used total body RNAseq data to estimate the fragments of mapped reads matching TE sequences (FPKM) for both novel and known TEs. Of the novel TEs, nine of 28 were expressed (Additional file 1: Figure S3, FPKM > 1), a similar proportion to that of the previously known TE families (49 of 116). Similarly, we found all 28 novel TE sequences and 114 known families had piRNAs generated against them (considering small RNA sequences 24-29 bp to be piRNAs). A subset of the piRNAs, those produced in the germline [7], are expected to show signatures of “ping-pong” amplification— small RNAs that match both sense and anti-sense strands of the TE sequence. These ‘ping-pong’ amplified sequences should also have a 10 bp overlap, a bias for uracil in the position 1 sense strand and adenosine bias at anti-sense position 10, due to their method of amplification [9, 54, 55]. We found that 60 elements (53 known families and 7 novel; 36 LTRs, 15 LINEs, 7 DNA transposons & 2 helitrons) showed signatures of ping-pong amplification (Additional file 2: Table S2) [56]. Similar proportions of novel and known elements showed ping-pong small RNAs (Additional file 1: Figure S3, Mann-Whitney U test W = 24, *p*-value > 0.1676). As expression is difficult to quantify for multicopy sequences, these measures of expression are mainly useful to show that the putative novel TEs have characteristics similar to those of the known TE sequences (Mann-Whitney U test W = 37, *p*-value > 0.05, Additional file 1: Figure S3), suggesting that they represent bona-fide TE sequences.

Unlike *D. pseudoobscura* and *D. persimilis*, there are no previous TE annotations for *D. miranda, D. lowei* and *D. affinis*. Most of the TE families we find in these species—57 of 77—are shared among all five species of the *D. pseudoobscura* group (these sequences were independently verified in each species, and considered to represent the same family based on ≥90% sequence identity, Additional file 1: Figure S1). We also find 20 additional TE families in these newly annotated genomes (Fig. 1b, Additional file 2: Table S2). These new annotations are likely to be incomplete: TEs may be missed in genomes assembled exclusively from short read data, particularly if there is missing pericentric heterochromatin [34], or due to our filtering removing valid TEs. Consistent with this, the estimated proportion of TE content is higher for nearly all TE orders in all species when estimated using *dnapipeTE* [57], which does not rely on genome assembly (Table 1), than compared to our reference based annotations. We limit our analysis of TE content, therefore, to the reliable annotations of TE families from *D. pseudoobscura* and *D. persimilis*. For analysis of horizontal transfer, we use only well-described TE families; note that these analyses only require that the presence of a TE be detected in genome.

### TE content in the *D. pseudoobscura* group genomes

We estimated overall TE content in the five sequenced species, and TE content broken down by order and family (Table 1, Additional file 2: Table S2). In particular, we quantified the TE content of our five focal species using five metrics: the proportion of the reference genome masked (using *RepeatMasker* [58]), the proportion of short reads mapping to each TE sequence, the proportion of short reads assembling to TEs using *dnapipeTE* [57], the number of insertions in each genome (called using *PopoolationTE2* [59], demonstrated across genomes in Additional file 1: Figure S2) and the estimated copy number of each TE family (Table 1 and Additional file 2: Table S2). We find a significant linear correlation between all metrics at the level of TE order, and most metrics at the level of TE family (Spearman’s rank correlation *p*-value < 0.00213), with one exception: We find no linear correlation between the estimated copy number of TE families and the proportion of the genome masked by that TE family (*p* > 0.58). In contrast to a previous study, which found similar proportions of LTRs and LINEs in the *D. pseudoobscura* genome [33], we find LTRs are more than twice as abundant as LINEs (Table 1) as seen previously [34]. It is worth noting an additional effort was put into finding novel LTRs in the putative TE set using *LTRHarvest* [60]*,* though downstream curation should have removed any false-positives.

Most of the TE content is due to the 57 TE families shared across the entire group (73–84% of insertions and 53–78% of each species reference TE content, Additional file 2: Table S2). Consistent with divergence in TE content between species over time, some TE families differ in copy number e.g. *HelitronN-1* in *D. miranda* makes up 1.1% of the genome, while it only constitutes 0.14% of the *D. lowei* genome (Additional file 2: Table S2). These differences are possibly due to stochastic expansion degradation/extinction of families over time, or differing fitness costs between species. Specifically, in the case of *HelitronN-1*, we collapsed together *HelitronN-1* and the closely related beneficial ISX sequence that has been co-opted for dosage compensation in *D. miranda*, as these have very similar sequences [61].

### Differences in TE content between species

**Fig. 2:**
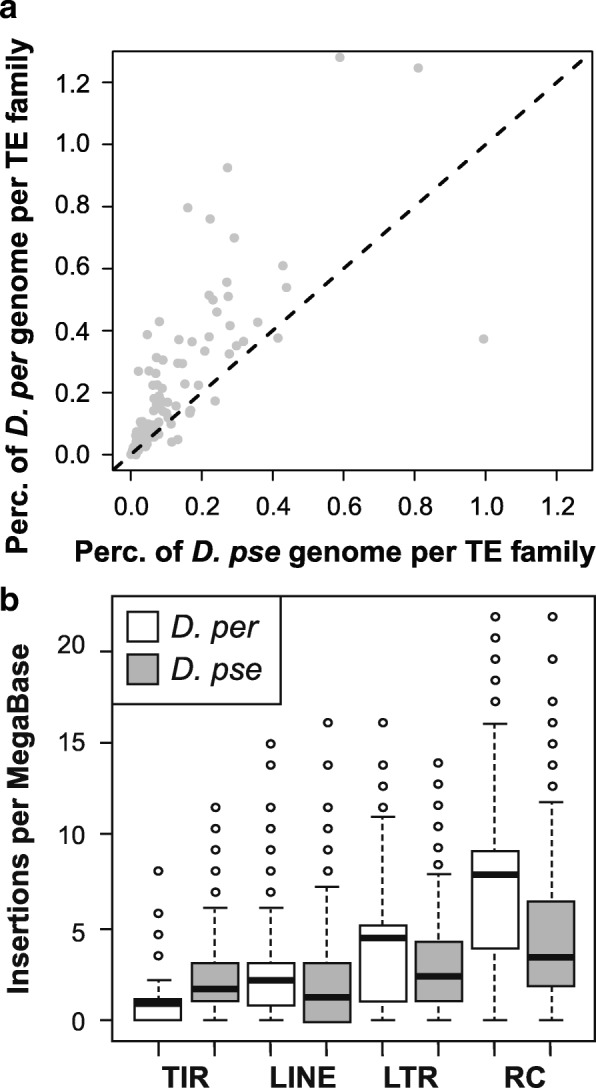
**a** Comparison of TE content of *D. persimilis* and *D. pseudoobscura* for each TE family. There is a significant correlation between copy number, but most TE families show more coverage in *D. persimilis* (GLM *t*-value = 23.532, *p*-value < 2e-16). **b** Density of insertions per MB for *D. persimilis* and *D. pseudoobscura* by TE order

At first glance, *D. persimilis* is an outlier in the group, with much higher TE content than the other species in the *D. pseudoobscura* group (Table 1). However, while we annotated the *D. miranda, D. lowei* and *D. affinis* genomes using a pipeline identical to that for other species, there is good reason to believe we may have underestimated the TE content of these species, as discussed above (Table 1, Additional file 2: Table S2). In any case, the *D. persimilis* reference genome does appear to have approximately double the TE content of *D. pseudoobscura*, which is likely well-annotated here (23.59 versus 12.33% for the reference genome, Fig. 2a, Table 1). This level of difference is perhaps surprising for these closely related species thought to hybridise in nature [45, 46, 62]. A previous annotation from the 12-genomes project also found a similar ~ 2-fold enrichment in TEs for *D. persimilis*, although the estimated TE content was lower than that found here (3 and 8% vs. 12.33 and 23.59% here) [33].

As these species share the same TE families, any difference must be due to a difference in copy number; in fact, we found higher copy numbers in *D. persimilis* for most TE orders (Figs. 1 and 2, Table 1, Additional file 2: Table S2), implying a 21.3Mbp larger genome size in *D. persimilis*. While it is true that *D. persimilis* has a larger genome than *D. pseudoobscura* ([63], the genomes of females of the two species are estimated to differ only by ~2Mbp, [64, 65]. The difference in copy number in the reference genomes may be partly due to the mixed male and female material used to construct the reference genomes. While considerable variation exists in *D. pseudoobscura* Y chromosome size between populations [41, 42], the *D. pseudoobscura* reference genome was likely generated from a strain containing the smallest Y chromosome type (Standard/Arrowhead, type V) [40, 41], and the *D. persimilis* from a strain with the most common *D. persimilis* Y, which is cytologically the largest Y-chromosome type in the two species [41]. Thus, the *D. persimilis* reference likely includes more TE-rich Y-chromosome sequence than the *D. pseudoobscura* one.

We therefore also estimated coverage from short read data which was collected exclusively from females and estimated the TE proportion with *dnapipeTE*. The bulk of the difference between *D. persimilis* and *D. pseudoobscura* seems due to a few families with large numbers of insertions in *D. persimilis* (e.g.*, Gypsy10_Dpse*, *HelitronN-1_Dpe*, *Gypsy17_Dpse*, and *MiniME_DP*; Additional file 2: Table S2)*.* Using these data, we estimate that *D. persimilis* has, at most, ~5Mbp more TE content than *D. pseudoobscura* (from copy number and *dnapipeTE* [57] estimates), consistent with the minor differences in genome size found between the females of the two species [65]. Any difference in TE content between the species may in part be mediated by indirect effects of the very large Y-chromosome in *D. persimilis*, as in addition to being themselves TE-rich, Y-chromosomes may have indirect effects on TE content: Y-linked variation in *D. melanogaster* and *D. simulans* has been shown to be associated with TE regulation [66], with Y chromosomes apparently driving poor TE regulation due to the increased heterochromatin load in the genome [66–68].

We asked if, as for other differences between the species, these number differences are enriched in the paracentric inversions fixed between *D. pseudoobscura* and *D. persimilis*. Outside these regions, genetic differences are relatively homogenized between the species, likely due to, while inside these regions, divergence accumulates due to reduced genomic exchange [46]. We find a mixed effect of the inversions on TE copy number differences. For LTRs, copy numbers in inversions are elevated in *D. persimilis*, as expected when compared to outside inversions (Fig. 2b, Additional file 3, using inversion windows defined in [69]; Mann Whitney U test: LTR insertions per MB inside inversions W = 53,686, *p*-value = 5.7e-05, near inversions W = 16,604, *p*-value = 0.113 and outside inversions W = 290,520, *p*-value = 0.141). But inversions have little effect on copy numbers of RC and LINEs, which occur at higher density in *D. persimilis* genome-wide (Fig. 2b, Insertions per MB, Mann Whitney U test: W > 335,780, *p*-value < 0.0001 for inside, outside and near inverted regions). These differences in the effect of inversion may reflect differences in timescales: LTR insertions tend to be young and highly polymorphic in *Drosophila* [70, 71], and thus should be affected by recent processes, such as post-speciation gene flow between these two species. We see no difference in TIR insertion densities (Fig. 2b, Insertions per MB, Mann Whitney U test: W > 150, *p*-value = 0.33).

### Evidence of recent recurrent transfer between species within the *D. pseudoobscura* group

Most TEs enter genomes vertically. Among those entering horizontally, a majority will enter from a closely related species in an overlapping range [11, 17–19, 67]. We looked at the rates of exchange of TE families between our focal species to assess the extent this contributes to the maintenance of active TE insertions. The geographic range of *D. pseudoobscura* overlaps with that of the other species studied here, apart from *D. affinis* and the subspecies *D. pseudoobscura bogotona* [72]. In addition, as mentioned previously, *D. pseudoobscura* and *D. persimilis* likely exchange genes through hybridisation, which is unlikely to occur in nature among the rest of the species in the group [45, 46].

Following Bartholome et al. [25], we compared silent divergence between species at TE sequences to those for genes [69]; *d*_*S*_ between species for recently horizontally transferred TE sequences will be low compared to that of vertically transmitted genes. To perform this analysis, we constructed a consensus sequence for each TE family for each species in the *D. pseudoobscura* group, and estimated synonymous site divergence (*d*_*S*_) using maximum likelihood [73] between these consensus sequences for each of the 10 species pairs.

**Fig. 3:**
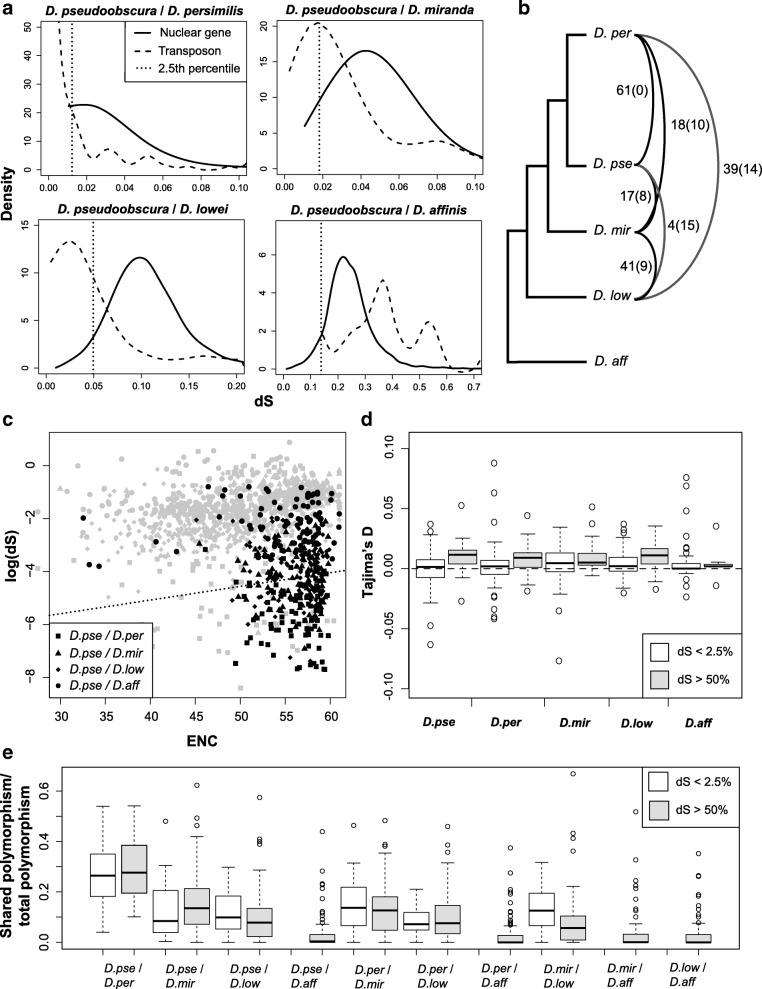
**a** Pairwise comparison of silent site diversity (*d*_*S*_) for nuclear genes (solid line) and shared TEs (dashed lines) between *D. pseudoobscura, D. pseudoobscura bogotana, D. persimilis* and other species. The lower 2.5% quartile for nuclear *d*_*S*_ is shown as the dotted vertical line. These distributions are consistent between all species pair comparisons (t-test *p*-value > 0.13), so only comparisons to *D. pseudoobscura* are shown. **b** The number of transfer events for transposable elements based on d_S_ and confirmed with VHICA. The number in brackets shows events that can be seen in the assembled phylogenies. Note that many events could be occurring between species vertically as well as horizontally. **c** effective number of codons (ENC) for genes (in grey) and TEs (black) versus *d*_*S*_ between species pairs. Each shape represents a species pair. The dotted line represents the lower 2.5th percentile per 5 EHC window for *D.pse/D.per* and *D.pse/D.mir* (due to high similarity)*.* These distributions are consistent between all species pair comparisons shown in Fig. 2a. (t-test *p*-value > 0.05), so only comparisons to *D. pseudoobscura* are shown. Again, only *D. affinis* shows no evidence of exchange between species. **d** Comparison of Tajimas D across species for frequently exchanged TEs and rarely exchanged TEs shows no difference, suggesting no population expansion. **e** Proportion of shared nucleotide polymorphism sites between TE sequences in species, out of total nucleotide polymorphism sites, divided by TE families with low *d*_*S*_ relative to nuclear genes and TEs with higher *d*_*S*_

We performed this analysis for 101 TE families (those with previously described coding sequences), except for comparisons with *D. affinis*, where we used the 39 of TE families with annotated coding sequence of the 57 TEs found in all species. We found a significant overall reduction in *d*_*S*_ for TEs compared to host genes for all species pairs (Fig. 3, Mann-Witney U test *p*-value < 0.05), excluding those involving *D. affinis* (Mann-Witney U test *p* = 0.23, comparisons to *D. pseudoobscura* shown in Fig. 3a). We find 76 of the 101 TE families (75.2%) with *d*_*S*_ that falls below that of the 2.5% quantile for nuclear genes in at least one comparison, suggesting potentially recent transmission between species (51 of 62 LTRs, 19 of 30 LINEs and 6 of 9 DNA transposons). Interestingly, 10 TE families meet the criteria for horizontal transfer between all species pairs excluding those involving *D. affinis* (*d*_*S*_ < 0.25% quantile: 1 TIR, 1 LINE and 8 LTRs), while 22 show no evidence of transfer (*d*_*S*_ > 50% quantile: 1 TIR, 1 helitron, 11 LINEs and 9 LTRs), suggesting that families differ in their propensity for transfer. For species comparisons of similar levels of divergence (e.g. *D. lowei-D. pseudoobscura*, *D. lowei-D. persimilis* and *D. lowei-D. miranda*), we find no significant difference between rates of exchange (Mann-Witney U test *p*-value > 0.08). In addition to horizontal transfer, selection on silent sites can also depress *d*_*S*_, providing an alternative explanation for the low *d*_*S*_ of TEs [74]. We therefore re-examined the *d*_*S*_ values using a method that controls for selection on silent sites due to selection on codon usage, *VHICA* (Vertical and Horizontal Inheritance Consistency Analysis) [74]. Consistent with our previous results, we find evidence of a high proportion of horizontally transferred families: 69 of 76 TE exchanges with low *d*_*S*_ are identified as horizontal exchanges with *VHICA* as well (*p*-value < 0.05, Fig. 3c, with 1 LTR, 5 LINEs and 1 DNA transposon identified as vertically transferred instead). We also looked specifically for evidence of exchange between non-sister taxa, which would result in gene-tree/species-tree discordance: of the 76 TE families with reduced divergence, phylogenies reconstructed for 42 are qualitatively inconsistent with the species tree (Figs. 1 and 3b). In principle, gene tree-species tree discordance could also be explained by incomplete lineage sorting, caused by TE sequences found in the common ancestor segregating into the descendant species such that TE with the most similar sequences are inherited by non-sister taxa. In this scenario, however, the TE consensus sequences should coalesce in the common ancestor, and thus would be expected to show high dS, not the low dS as seen here.

Transfer across species boundaries, in addition to happening by largely unknown mechanisms of horizontal transfer [20], can also occur via hybridisation. Within the *D. pseudoobscura* subgroup, *D. pseudoobscura* and *D. persimilis*, can produce fertile hybrids with others to some degree and are likely to regularly exchange genes in nature; (Machado et al. 2007), therefore, we cannot determine if these apparent transfer events are true horizontal events or hybridisation followed by introgression of TEs, but not genes. In fact, we do not find more evidence of transfer for LTRs and TIRs than LINEs, as is typical for genuine cases of horizontal transfer [20, 25], suggesting hybridisation as a mechanism of TE transfer in this group. That said, there is still ample evidence of exchange of TE families between species pairs that are sympatric [37, 47, 48, 75], but which cannot hybridise, e.g.*, D. pseudoobscura*-*D. lowei, D. pseudoobscura*-*D. miranda* and *D. miranda-D. lowei* (Fig. 3b), and reduced *d*_*S*_ between these species and *D. pseudoobscura* for TEs compared to genes (Fig. 3a, c).

In contrast, exchanges with the allopatric species in the group, *D. affinis*, there is little evidence of exchange, consistent with geographic isolation limiting opportunities for transfer (Fig. 3a, Mann Whitney U test: *p <* 3.5e-08, Additional file 2: Table S4). For TEs, *d*_*S*_ between other focal species and *D. affinis* was significantly higher than for host genes (Fig. 3a, b). Additionally, we find no signatures of TE exchange using *VHICA* (Fig. 3c). Thus, while we see abundant transfer between species that are sympatric, but not appear able to hybridise, the absence of exchange with *D. affinis* suggests routes of horizontal transfer that depend on proximity.

Under some scenarios, true horizontal transfer events allow TEs to escape host silencing, and are thus followed by bursts of transposition [9], which eventually subside as host silencing strengthens. We examined the TE sequence data for signatures of such bursts. For one, copy number expansion following horizontal transmission should be evident by an excess of low frequency single nucleotide differences between TE sequences, and thus a negative value of Tajima’s D [25, 76]. We estimated Tajima’s D for each TE family in each species; in almost all cases, Tajima’s D not significantly below zero (significance obtained from simulation [77]), suggesting no recent expansion in copy number (Fig. 3d). In contrast to results for the *D. melanogaster* group showing copy number expansion [25]. Interestingly, Tajima’s D is slightly, non-significantly, lower in highly exchanging TE families compared to rarely exchanging TE families (Fig. 3d, t-test *p* > 0.57). Similarly, bursts of transposition would be expected to yield variation in copy number between species. In fact, we do see large variation in copy numbers for each family across species (Table 1, Additional file 2: Table S2). However, we compared the coefficient of variation, for pervasively transferring TEs, non-transferring TEs and all other TEs to ask if HT is associated with the differences in copy number between species. We find no difference in the coefficient of variation of copy number for pervasively transferring families and non-transferring families (Mann-Whitney U test, *p* > 0.19 for all comparisons).

As an alternative to the horizontal transfer followed by burst model, these species may instead exchange TEs constantly (either via hybridisation or otherwise), so that any bursts following transfer are moderated. While previously we looked for a lack of divergence, further evidence for rampant horizontal exchange would be shared nucleotide polymorphisms across species boundaries, suggesting multiple TE sequences have been shared between species as opposed to a single shared copy (Fig. 3e, Additional file 2: Table S5). This suggests recurrent transmission between species, rather than single events [25, 76], or polymorphisms inherited from the TE in a common ancestor. Alternatively, there is less constraint on polymorphism in transposable elements, allowing recurrent mutation and polymorphisms to drift to higher frequencies in shorter periods of time following their horizontal acquisition. Generally, we find a negative correlation between synonymous divergence and shared polymorphism (significant for all comparisons apart from with *D. affinis, p* < 0.05, no negative correlation between *D. persimilis/D. pseudoobscura*, Additional file 1: Figure S6).

These results together are likely due to a combination of gene flow between species in the wild, and recurrent horizontal transfer via other, as yet unknown, mechanisms, as in [11, 78, 79]. TE transfer following hybridisation may result in more homogenisation of TEs than of genes: while introgressed genes may be purged due to hybrid incompatibilities or genetic drift, their linked TEs may transpose readily after hybridisation, becoming unlinked from the introgressed genes. Particularly if accompanied by small RNA suppressors, these TE variants may be maintained in the new host with no accompanying change in Tajima’s D [9]. Further, recurrent horizontal transfer between non-hybridising sympatric species of the *pseudoobscura* subgroup may recur frequently enough that TE families are freely shared between each of the species pairs, resulting in not only low *d*_*S*_, but shared polymorphism and lack of copy number expansion (Additional file 1: Figure S6).

## Conclusion

Like *D. melanogaster*, the *D. pseudoobscura* group shows highly active TEs that appear to be constantly undergoing a cycle of acquisition, expansion and high activity, suppression and finally extinction [4]. Surprisingly, despite TE exchange between species, the group shows distinct differences in TE content and TE densities consistent with high activity and turnover. Some of these differences may due to differences in quality of assembly of each species genome and method used to identify TE insertions. We find a distinct expansion in TE numbers in *D. persimilis* potentially due to differential regulation of TEs. Overall this suggests that despite frequent gene flow, TE dynamics can evolve rapidly across the lifetime of a TE family.

Due to the history of the first recorded instance of a horizontal transfer of a transposable element [12, 80, 81], we previously thought these transfers are rare and likely catastrophic events. However, an expanding body of evidence suggests that these events are likely a common occurrence throughout genomes, becoming more and more common the more closely related two species are [17, 25]. This transfer of elements is possibly even recurrent in some cases*.* Our results support the view that the TE content of genomes is fluid, with TEs moving between genomes easily, with only occasional catastrophic events such as the invasion of the *P*-element.

## Methods

### Sequence data

We used publicly available reference genomes for five species: *D. pseudoobscura* (NCBI: PRJNA18793)*, D. persimilis* (NCBI: PRJNA29989 genome assembled from Sanger sequence reads, http://popoolation.at/persimilis_genome/ for the genome based on illumina reads)*, D. affinis* reads from (NCBI: ERX103526) and assembly (http://popoolation.at/affinis_genome/), *D. lowei* (http://popoolation.at/lowei_genome/; Palmieri et al. 2014), *D. miranda* (NCBI: PRJNA77213) All sequence data used is summarized in Additional file 2: Table S1. We also used publicly available paired-end illumina data from inbred lines for four of these species [*D. persimilis* (SRA: SRR330426), *D. miranda* (SRA: SRR1925723), *D. lowei* (SRA: SRR330416 and SRR330418) and *D. affinis* (ENA: ERR127385)]. As we were unable to find publicly available paired-end illumina data for *D. pseudoobscura*, we used a data generated from an individual wild *D. pseudoobscura* made homozygous for the reference third chromosome inversion type (SRA: SRR617430; Fuller et al. 2016). As a result, only the third chromosome represents a wild chromosome, the rest of the genome is a mosaic of material from the wild and from the two different balancer stocks used.

### De novo annotation of transposable elements in the *D. pseudoobscura* group

We annotated TE families in all five species, as well as putative TE sequences in the more diverged species (such as *D. lowei* and *D. affinis*), and compared our de novo annotations to the previous annotations for *D. pseudoobscura* and *D. persimilis*. These sequences were identified using *RepeatModeler* and *LTRHarvest* [60, 82]. Due to potential false positives called by these tools, we further filtered sequences, as outlined in Additional file 1: Figure S1 to give us a set of ‘high confidence’ TE annotations confirmed across several species.

To de novo annotate the transposable elements, as shown in Additional file 1: Figure S1:

1. We recovered a set of TE candidates for each species using the reference genomes. We used two separate pipelines: *(i) Repeatscout* and *PILER* in the *RepeatModeler* pipeline (default parameters) [82, 83], with all sequences designated as microsatellites and simple repeats removed from the output, and *(ii) LTRHarvest*, which finds LTR retrotransposons (using parameters recommended in the *LTRHarvest* manual*:* -tis *-suf* -lcp -d*es -sds –dna;* −seed 10*0* -minlenltr 100 -maxlenltr 1000 -mindistltr 1000 -maxdistltr 15,000 -xdrop 5 -mat 2 -mis − 2 -ins − 3 -del − 3 -similar 90.0 -overlaps best -mintsd 5 -maxtsd 20 -motif tgca -motifmis 0 -vic 60 -longoutput) [60]. Though this step may bias us to find primarily LTRs, we note that most previously known TEs we find are LTRs, while most (19 of 41) novel elements are DNA transposons (Additional file 2: Table S2).
2. Step 1 resulted in a set of 769 candidate TE sequences, ranging from 208 bp to 14.5 kb. We used BLAST to filter and annotate the candidate TEs (parameters: e-value <1e-08, −word_size 10, −perc_identity 85) [84], by searching a database of all known *Repbase* and *Flybase* transposable element sequences for *Diptera* (including 121 TEs previously found in *D. pseudoobscura, D. persimilis* or *D. miranda*), with sequenced duplicated between the data bases removed using a custom python script.
  - Sequences that show single BLAST hits (e-value ≤1e-08) to this data base were assumed to represent a previously identified TE family. We discarded these sequences and used the Repbase/Flybase TE sequence to represent the family instead. (349 sequences).
  - From the remaining sequences, those that showed BLAST hits to several TE families, all from one superfamily, were considered to potentially represent a previously unidentified family within that superfamily. (180 sequences).
  - Of the remaining sequences, those with hits all in a single order, but to multiple superfamilies, were potentially novel TEs within this order. (18 sequences).
  - For sequences which had no potential TE family assigned in Step 2 (222 sequences), we attempted to find matches by aligning them to the online NCBI non-redundant database using megablast. Of these, 202 had annotated or predicted genes as the primary BLAST hit; these were discarded. The remaining potentially novel TEs were retained (20 sequences),
3. To facilitate downstream analysis, we obtained a single representative sequence for the potential novel TEs identified in Steps 2b, c and d, as is already done for those in Step 2a. To do this, we clustered sequences found for all species using *vmatch* (recommended *LTRHarvest* parameters: -dbcluster 95 7 -p -d -seedlength 50 -l 1101 -exdrop 9) [85]. We confirmed these clusters by BLASTing novel TE sequences to themselves and grouping them by similar matches (parameters: e-value < 0.00001, −word_size 10).
4. As these may only represent partial TE sequences, we further assembled the grouped sequences using *Trinity* (default parameters) to collapse similar sequences and get a representative sequence for the cluster, even if only a fragment of the consensus sequence [86]. We checked these assemblies and clusters by aligning sequences from the cluster and with the *Trinity* assembly (if applicable) using *MAFFT* (parameters: --thread 3 --threadit 0 --reorder --leavegappyregion –auto) [87], to ensure that the assembly or longest sequence representing the putative novel TE was recovered. From each cluster of similar sequences, we took the longest sequence as the representative fragment of each putatively novel family.
5. Some of the putatively novel families identified in 2b may instead be divergent representatives of known families. To see whether this was the case, we again attempted to identify previously known families among them using the consensus sequences from the five species genomes. We aligned novel TEs pairwise to all *Repbase* TEs using *MAFFT* (parameters: --thread 3 --threadit 0 --reorder --leavegappyregion --auto) and used a custom *python* script to find the number of diverged aligned bases. We defined sequences as belonging to a known family if they were > 90% similar to a known family across the sequence, following [51]. Two families of the novel sequences were found to belong to known families in this way (an I-element and a Jockey element), but were closely related to insertions in distant relatives of the *obscura* group (*I*-4_DF from *D. funebris* and *Jockey*-8_DRh from *D. rhopaloa,* respectively). We therefore retained these sequences in our data set, as they likely represent diverged copies of these families, or ancient horizontal acquisitions.
6. From Steps 1–5, we found 567 candidate TE sequences, 349 of which belong to previously described TE families, including all 121 families previously found in the *D. pseudoobscura* group (‘known’ families), and 446 others (putative ‘novel’ families). We proceeded to filter sequences from this set which were represented by very few or very short matches to the reference genomes.
  - First, we used the 567 sequences to repeat mask the reference genome of each species using *RepeatMasker* (parameters: –no_is –norna –no_low –gff –gccalc –u –s –cutoff 200) [58], following recommendations in [70]. We required that the families have at least 25 Repeatmasker hits in at least one species (237 sequences retained, 116 known and 121 novel families).
  - We then estimated the copy number of each TE family for each species from the Illumina short read data from adult females, discarding those estimated to have a median coverage less than 2-fold that of the third chromosome for less than 80% of the length of the sequence. To do this, we mapped short reads to the repeated masked reference genome and the 237 TE sequences retained from the previous step using BWA MEM (parameters: paired end –t 5 -M) [88], and estimated coverage with *bedtools genomecov* [89]. Due to the poor assembly of the *D. persimilis* genome, we used a reference consisting of the *D. pseudoobscura* genome and the *D. persimilis* TE sequences. (157 sequences retained, from 116 known and 41 families novel to this species group).

We considered these 157 sequences to be an adequate representation of the TE content in the *pseudoobscura* group, though we recognize that our conservative approach may have discarded some true TE sequences.

Using this method, we found strong support for 114 of the 121 TE families previously described in *D. pseudoobscura, D. persimilis* or *D. miranda* and 2 TEs previously identified in other *Diptera* species. We found 41 putatively novel sequences, including two subfamilies of previously known sequences, 30 newly assembled sequences which BLAST exclusively to one super family, and nine potentially new families that BLAST to one TE order. We also found 15 sequences that cannot be assigned an order (either due to BLAST hits to multiple orders, or no BLAST hits). These 15 sequences passed all filters, including being found multiple times in species genomes and did not correspond to genes or other NCBI sequences in a non-redundant BLAST search. To avoid unreliable inferences, we discarded these sequences from downstream analyses, but gave each of the 41 novel sequences an ID (Additional file 2: Table S2), and included them in masking and mapping stages. Sequences are available in Additional file 4.

For an independent verification of TE content, unbiased by reference genome, we generated dnapipeTE [57] profiles for each species using illumina sequencing information (−genome_coverage 0.5 –sample_number 2 –genome_size previously estimated size). We compared the proportions of each TE order in the genome to our referencegenome estimates and the proportion of reads mapping to TE sequences.

### Estimating TE density in the reference genome

We used *RepeatMasker* v. 4.0.6 to mask each reference genome using the 157 consensus TE sequences and 15 unknown sequences from the de novo annotation, (parameters: –no_is –norna –nolow –gff –gccalc –u –s –cutoff 200) [58]. To estimate the TE density of each genome, we calculated the density of TE bases per 1 MB sliding window (with a step size of 100 kb, after removing all N bases [e.g. TE bases / [window size – Ns in chromosome]]), across both assembled scaffolds and unassembled contigs from each reference genome.

### Identifying insertions in reference genomes and in sequenced third chromosome lines of *D. pseudoobscura*

To identify insertion sites in the reference genomes of *D. pseudoobscura* and *D. persimilis*, we used the *PopoolationTE2* pipeline [90]. We chose to use the *D. pseudoobscura* masked reference, rather than the fragmented *D. persimilis* reference, as it facilitated mapping reads to genomic insertion sites. We expect similar results as these species are closely related (0.018 average synonymous divergence [45]), and we find that a similar proportion of reads map to TEs regardless of whether the *D. pseudoobscura* or *D. persimilis* genome is used (27.63 vs 27.27%).

We then mapped available Illumina reads to the repeat masked references, the consensus TE sequences, and to sequences matching these consensus TEs identified by *RepeatMasker* using BWA-MEM (parameters: paired end –t 5 -M, with secondary alignments reported, but marked) [88]. Using masked TE sequences to aids mapping of degenerate TE sequences, as described in [90].

Following mapping, we generated a ppileup file summarizing identities and locations of TE insertions for all lines in *PopoolationTE2* (default settings, −-map-qual 10) and subsampled to a physical coverage of 25, removing secondary alignments. As these sequences are mostly from inbred lines, we required the estimated frequency to be at least 50% (default parameters, −-target-coverage 25, −-min-count 5, minimum frequency = 0.5) [90]. We then identified the number of insertions per MB window (after adjusting for the number of N bases in the window [e.g. TE number / [window size – Ns in window]]) across the genome of each species.

### Expression confirmation of putative TE sequences

To see which TEs showed evidence of expression, we used RNAseq data for mRNAs (SRA: SRR1956914, taken from [52]) and small RNAs (SRA: SRR032435, taken from [91]) from the *D. pseudoobscura* reference line (MV-25). Before further analysis, we trimmed all genomic and RNAseq Illumina reads used with *Sickle* to remove low quality sequence data (default parameters for long reads, minimum length = 16 for small RNAs, 50 for mRNAs), and removed reads that were unpaired (apart from the small RNA reads) after this step from the sequence data [92].

We mapped small RNA sequences from *D. pseudoobscura* to known and novel TEs identified in that species, using publicly available small RNA reads from the reference strain ([91], SRA: SRR032435). We first removed non-TE related small RNAs, following [7, 93], by mapping to a database of known *Drosophila* viruses and small RNAs other than those that are TE-related, including miRNAs, viral siRNAs, snoRNA [93], using *BWA aln* and allowing for up to 3 mismatches (parameters: -n 3) [7, 88]. We then mapped the remaining reads to the repeat masked *D. pseudoobscura* reference genome and the novel and known TE sequences identified in this study (*BWA aln* parameters: -n 3, maximum 2 alignments). We classified small RNAs by length and orientation using a custom python script and the *Pysam* python library, following [94]. Specifically, we considered small RNAs from 21 to 23 to be siRNAs and from 24 to 29 to be piRNAs [95]. We used *bedtools* (*intersect*, −wa –wb –f 0.3 –r), to check for a 10-bp overlap between sense and anti-sense matches and used *sequence logos* [56] to check for the 1-T, 10-A bias, both associated with ping-pong amplification, a characteristic feature of piRNAs [96].

### Detecting short range horizontal transfer events within the pseudoobscura group

To detect horizontal transfer of TEs within the five species examined, we compared divergence between consensus TE sequences to genomic divergence, following the rationale described in [25]. We limited this analysis to families found in at least 3 species and with an annotation on Repbase. As this method requires consensus sequences, we constructed these for each TE family and each species, we identified the major allele for each species at each variable site using *GATK v3.5–0-g36282e4 HaplotypeCaller*, with ploidy levels set to the estimated copy numbers based on coverage of the TE sequence, and using *FastaAlternateReferenceMaker* (default parameters) to generate fasta sequences from the mapped data [97]. We aligned these consensus sequences from each species using *MAFFT* (parameters: --thread 3 --threadit 0 --reorder --leavegappyregion –auto) [87] and generated a phylogeny of each sequence using the *Repbase* annotation and *PhyML* (parameters: -M GTR) [98]. We obtained a total of 39 annotated alignments that included sequences for *D. affinis* comparisons, and 62 additional sequences for all other *pseudoobscura* group species comparisons (noted in Additional file 2: Table S2).

We estimated synonymous site divergence (*d*_*S*_) in the TE sequences pairwise between species using *codeml* (with transition–transversion rates estimated from the data, and codon frequencies from the nucleotide frequencies) and the coding regions for these TEs as annotated in *Repbase* [51, 73]*.* We then compared *d*_*S*_ of TEs to that of orthologous genes between species obtained in the same way, taken from Avila et al. (2014). Following Bartolomé et al. (2009), we considered an individual family to show strong evidence of exchange if its *d*_*S*_ value was below the 2.5% quantile of the *d*_*S*_ of all nuclear genes, to have potentially transferred if *d*_*S*_ was between the 2.5 and 50% quantiles, and to show no evidence of transferring if above the 50% quantile.

We used the VHICA pipeline to confirm these putatively horizontal transfers, estimating the effective number of codons for each TE coding sequence and gene sequence for each species using the VHICA R package [74]. We considered a TE to have horizontally transferred with a significant *p*-value from the VHICA R package and a *d*_*S*_ below two standard deviations of the genic mean, per ENC [74].

We examined polymorphism within TE families for evidence of horizontal transfer. We estimated Tajima’s *D* of each TE using *Popoolation* [99],with the TE copy number as the sample size. As negative Tajima’s D may reflect recent expansion of a TE family [25]. We compared the levels of polymorphism shared among TEs in each species between potentially transferred TEs (*d*_*S*_ < 2.5% quantile) and TEs that are unlikely to have transferred (*d*_*S*_ > 50% quantile). Using known estimates of Watterson’s theta for *D. pseudoobscura,* we calculated the expected neutral distribution of Tajima’s D for 1000 simulations in *ms* [77].

## Additional files

Additional file 1: **Figure S1.** Pipeline for TE annotation. **Figure S2.** TE density across the genomes of each species, found using *PopoolationTE2,* sorted by TE order. **Figure S3.** Comparison between putatively novel and known TE sequences for (A) length, (B) expression, (C) small RNA silencing expression and (D-F) copy number. **Figure S4.** Distribution of TE copy numbers per species. **Figure S5.** Phylogenies of each TE super family including novel TE families, used to calculate patristic distances. **Figure S6.** Correlation between silent substitutions in TEs between species and the proportion of silent shared polymorphism between species. (DOCX 1531 kb)

Additional file 2: **Table S1.** *D. pseudoobscura* species group lines used in this study. **Table S2.** TEs found in *D. obscura* group. Sorted by if they are previously discovered or novel, then by Order and super family. Transmission states if the TE family is found to transfer between species. **Table S3.** Diagonal table showing the total number of TE families found in each species for comparison. In brackets, the number of novel TE families found shared between species. **Table S4.** Comparisons of dN/dS between TEs and nuclear genes. The dS values presented here are compared to the dS values of nuclear genes between the given species calculated previously. We considered a transfer event between two species to have occurred if the TE dS value is less than the 2.5th percentile for nuclear genes. For instances where no dS for nuclear comparisons are available, we used the dS between *D. pseudoobscura* and the species of interest. **Table S5.** Number of unique and shared polymorphic sites for each species comparison, for each TE family, used in the boxplots in Fig. 2e. (XLSX 153 kb)

Additional file 3: TE insertion density per megabase (estimated from PopoolationTE2 output) for each TE order and each species analysed here. (TXT 166 kb)

Additional file 4: Fasta file of TE sequences generated in the TE annotation, with basic description of each TE sequence. (TXT 892 kb)

## Abbreviations

FPKM: Fragments per kilobase of gene per million reads
LINE: Long interspersed nuclear element
LTR: Long terminal repeat
RC: Rolling circle
TE: Transposable element
TIR: Terminal inverted repeat
