# Supplementary material for: Extensive exchange of transposable elements in the Drosophila pseudoobscura group
Source: Mob DNA. 2018 Jun 19;9:20. doi: 10.1186/s13100-018-0123-6 (PMC6006672; doi:10.1186/s13100-018-0123-6)
Supplement: Supplementary file 1 — Figure S1. Pipeline for TE annotation. Figure S2. TE density across the genomes of each species, found using PopoolationTE2, sorted by TE order. Figure S3. Comparison between putatively novel and known TE sequences for (A) length, (B) expression, (C) small RNA silencing expression and (D-F) copy number. Figure S4. Distribution of TE copy numbers per species. Figure S5. Phylogenies of each TE super family including novel TE families, used to calculate patristic distances. Figure S6. Correlation between silent substitutions in TEs between species and the proportion of silent shared polymorphism between species. (DOCX 1531 kb) [file 13100_2018_123_MOESM1_ESM.docx]

### Figure S1: Pipeline for TE annotation.

###

### Figure S2: TE density across the genomes of each species, found using *PopoolationTE2,* sorted by TE order.

###
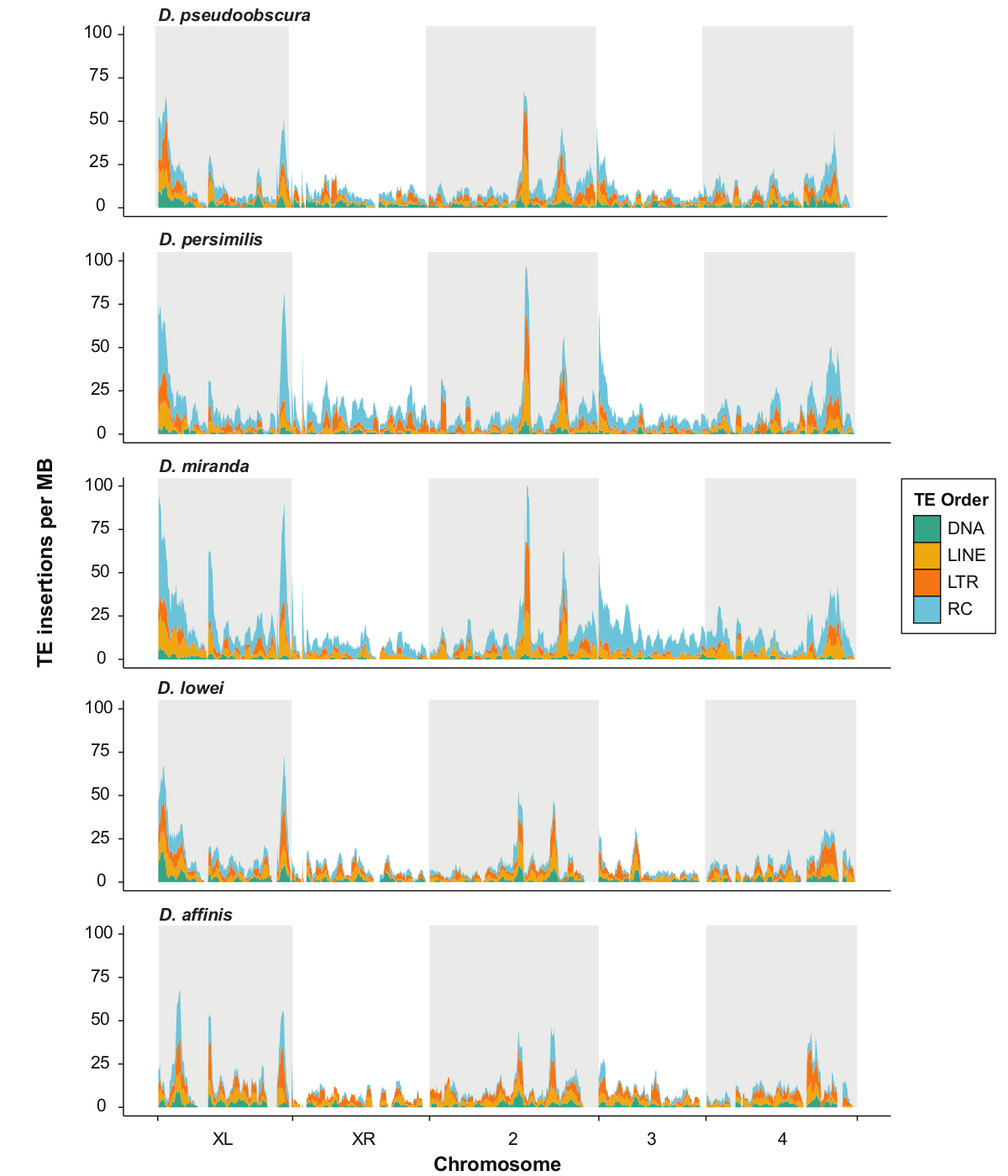


### Figure S3: Comparison between putatively novel (grey) and known TE sequences (white) for (A) length, (B) expression, (C) small RNA silencing expression and (D-H) copy number.

###

**Figure S4:** Distribution of TE copy numbers per species.


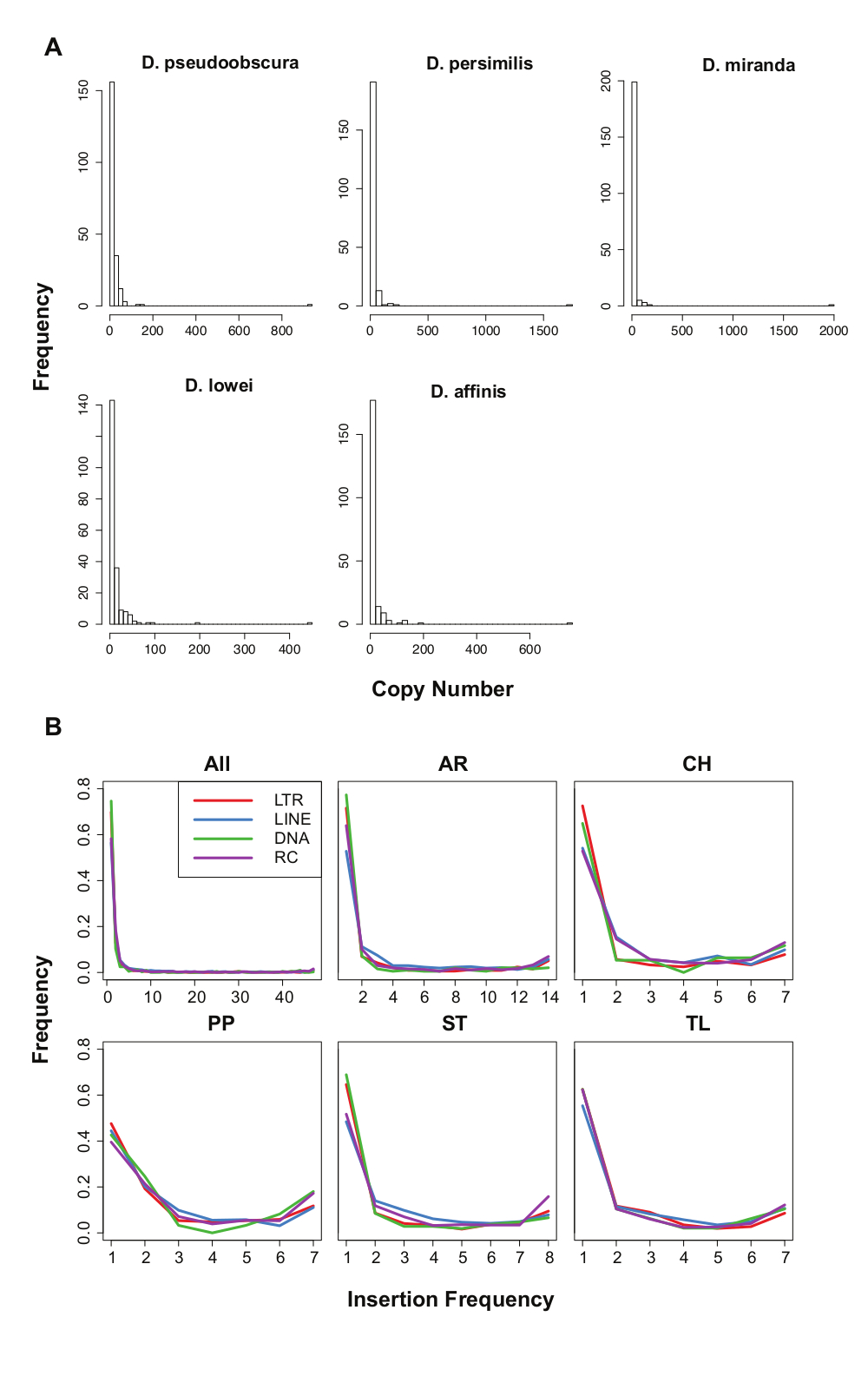


**Figure S5:** Phylogenies of each TE super family including novel TE families, used to calculate patristic distances.

**
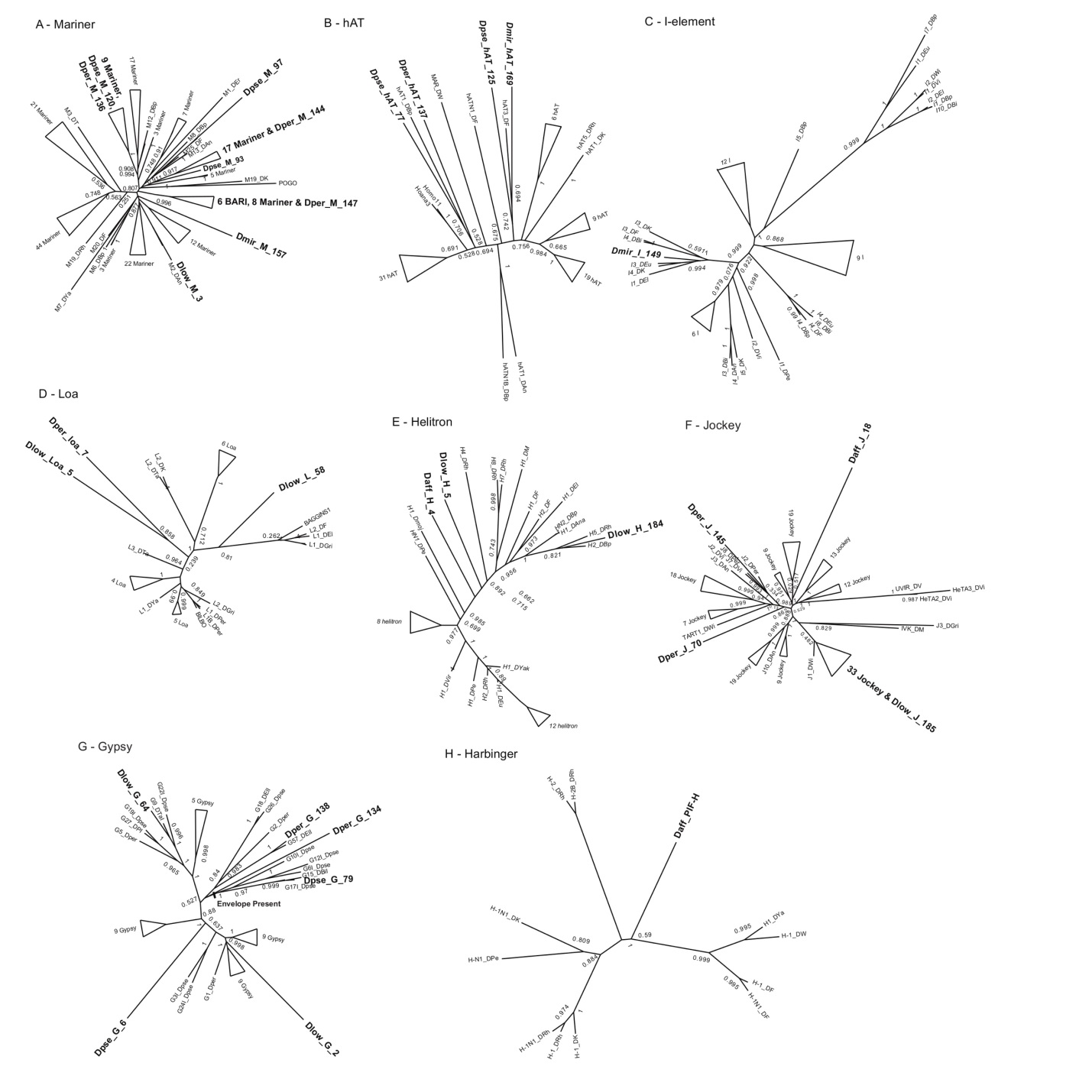
**

### Figure S6: Correlation between silent substitutions in TEs between species and the proportion of silent shared polymorphism between species.
